# Diagnosis of Chronic Granulomatous Disease: Strengths and Challenges in the Genomic Era

**DOI:** 10.3390/jcm13154435

**Published:** 2024-07-29

**Authors:** Conor J. O’Donovan, Lay Teng Tan, Mohd A. Z. Abidin, Marion R. Roderick, Alexandros Grammatikos, Jolanta Bernatoniene

**Affiliations:** 1 Department of Paediatric Immunology and Infectious Diseases, Bristol Royal Hospital for Children, University Hospitals Bristol and Weston NHS Foundation Trust, Upper Maudlin Street, Bristol BS2 8BJ, UK; 2 School of Cellular and Molecular Medicine, University of Bristol, University Walk, Bristol BS8 1TD, UK; 3 Department of Paediatrics, University Malaya Medical Center, Lembah Pantai, Kuala Lumpur 59100, Malaysia; 4 Department of Paediatrics, Faculty of Medicine and Health Sciences, Universiti Putra Malaysia, Serdang 43400, Malaysia; 5 Department of Immunology, Southmead Hospital, North Bristol NHS Trust, Bristol BS10 5NB, UK

**Keywords:** inborn errors of immunity (IEI), chronic granulomatous disease (CGD), NADPH oxidase, nitroblue tetrazolium (NBT), dihydrorhodamine (DHR), genetic testing, genomic testing, screening

## Abstract

Chronic granulomatous disease (CGD) is a group of rare primary inborn errors of immunity characterised by a defect in the phagocyte respiratory burst, which leads to severe and life-threatening infective and inflammatory complications. Despite recent advances in our understanding of the genetic and molecular pathophysiology of X-linked and autosomal recessive CGD, and growth in the availability of functional and genetic testing, there remain significant barriers to early and accurate diagnosis. In the current review, we provide an up-to-date summary of CGD pathophysiology, underpinning current methods of diagnostic testing for CGD and closely related disorders. We present an overview of the benefits of early diagnosis and when to suspect and test for CGD. We discuss current and historical methods for functional testing of NADPH oxidase activity, as well as assays for measuring protein expression of NADPH oxidase subunits. Lastly, we focus on genetic and genomic methods employed to diagnose CGD, including gene-targeted panels, comprehensive genomic testing and ancillary methods. Throughout, we highlight general limitations of testing, and caveats specific to interpretation of results in the context of CGD and related disorders, and provide an outlook for newborn screening and the future.

## 1. Introduction

Chronic granulomatous disease (CGD) represents a group of rare and complex primary inborn errors of immunity, whose common pathology is a significant deficit in the respiratory burst pathway in phagocytes, recently reviewed in [1,2,3,4,5]. Our understanding of CGD pathogenesis, diagnosis and management has improved significantly since early studies, which identified CGD as a functional disorder of phagocytes [6,7,8,9,10,11]. Despite these advances, it remains challenging to achieve a timely and accurate clinical diagnosis of CGD. The current review, informed by our modern understanding of CGD in its molecular pathophysiology, aims to provide a comprehensive overview of the diagnosis of CGD and associated challenges, with an up-to-date focus on functional assays, molecular and genetic testing.

## 2. Clinical Features and Inheritance

CGD is felt overall to be a rare disease, with varied reported estimates of prevalence, which differ geographically and by population rate of consanguinity [11,12,13,14]. In Western Europe and the US, prevalence is reported as 1 in 200,000–250,000 [15,16]. The clinical spectrum of CGD varies in terms of disease manifestations, severity and age at presentation, which presents a challenge to recognition and diagnosis. Severe cases (predominantly X-linked recessive in inheritance), tend to present early at 9–14 months of age, and are diagnosed early at 2.1–4.9 years [13,15,17,18]. Milder cases (predominantly autosomal recessive) tend to present later at 2.5–3.4 years, and are also diagnosed later at 5.8–8.8 years [13,15,17,18]. Diagnoses in early infancy, adolescence and adulthood are unusual, but occur increasingly due to increased awareness and uptake of testing [11,19,20,21,22,23].

CGD is characterised by four major clinical features, reviewed in [1,2,11,24,25,26,27,28,29]: (1) predisposition to infection by a classically described subset of catalase-positive bacteria and fungi; (2) development of tissue granulomas in respiratory, gastrointestinal and genitourinary tracts, which may lead to local complications; (3) predisposition to inflammatory bowel disease (IBD), which may be very early in onset and severe; and (4) other autoinflammatory or autoimmune complications. The overall incidence and severity of infective complications is highest in the first decade of life, and lower thereafter [30]. Most pathogens that cause infection in CGD patients also cause sporadic infection in immunocompetent hosts (e.g., *Staphylococcus aureus*, *Serratia marcescens*, and *Burkholderia cepacia* complex), but infections in CGD patients are more likely to be severe and frequent [30,31]. Virulence determinants other than catalase are also likely to be essential to virulence in CGD, as reviewed in [32], and evidenced by mouse models and clinical data [33,34,35,36]. Children with CGD are more likely to experience failure to thrive, organ dysfunction, poor quality of life, and increased risk of secondary inflammatory complications, such as haemophagocytic lymphohistiocytosis (HLH) [37,38,39,40]. Prior to the advent of modern therapies, most children with CGD died before 10 years of age [8], and infection, particularly invasive fungal infection, remains the leading cause of death [17,30,41,42]. Mortality risk is influenced by organ dysfunction [43], and the degree of detectable residual respiratory burst activity [44].

CGD can occur sporadically, but also demonstrates inheritance in X-linked recessive (XR) and autosomal recessive (AR) patterns. XR disease is most common worldwide, predominantly affecting males, and rarely females with very skewed X-chromosome inactivation (XCI) [12,17,40,45,46]. AR CGD has equal sex distribution, and is more common in consanguineous populations, surpassing XR CGD in prevalence in some countries [47,48,49]. There is also a well-described carrier phenotype for haploinsufficient female carriers of the XR mutation, characterised by predisposition to autoinflammatory and autoimmune phenomena, and milder infective and IBD-like symptoms [50,51]. Indeed, some argue that a subset of haploinsufficient carriers should be managed akin to *bona fide* CGD cases [45,50,52].

## 3. Molecular and Cellular Pathophysiology

**Figure 1:**
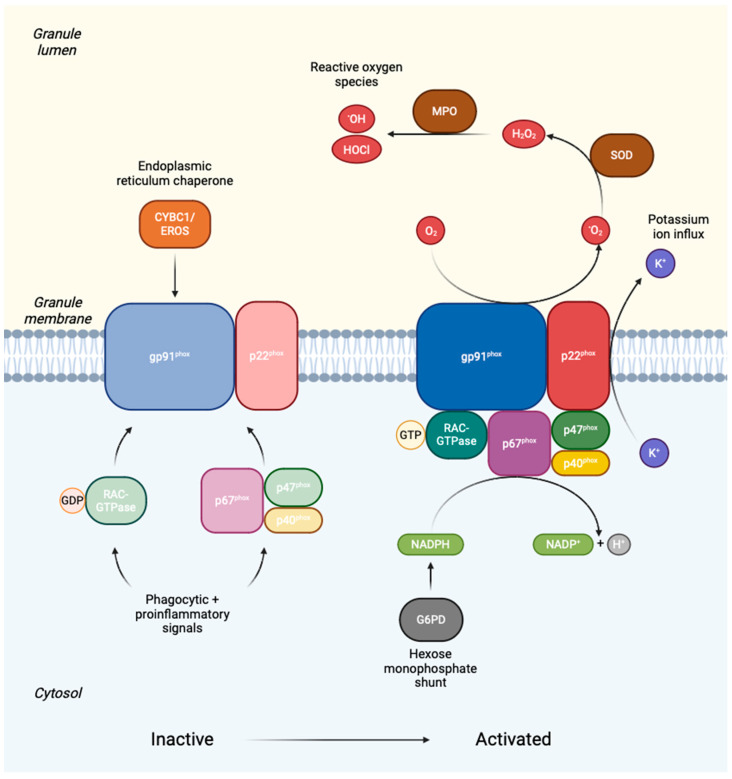
Factors required for expression, activation and function of NADPH oxidase. CYBC1/EROS cytochrome b_558_ chaperone 1, G6PD glucose 6-phosphate dehydrogenase, GDP guanosine diphosphate, gp91^phox^/p22^phox^/p67^phox^/p40^phox^/p47^phox^ NADPH oxidase core enzyme components, GTP guanosine triphosphate, H^+^ hydrogen ion, H_2_O_2_ hydrogen peroxide, HOCl hypochlorous acid, K^+^ potassium ion, MPO myeloperoxidase, NADPH/NADP nicotinamide adenine dinucleotide diphosphate, O_2_ molecular oxygen, ·O_2_ superoxide radical, ·OH hydroxyl radical, RAC2 Rac family small GTPase 2, SOD superoxide dismutase. Figure made with BioRender^®^.

All forms of CGD share a common pathology: failure of phagocytes (primarily neutrophils, monocytes and tissue macrophages), to produce reactive oxygen species (ROS) in the respiratory burst, reviewed in [53]. Central to this pathway is the 5-subunit enzyme complex nicotinamide adenine dinucleotide phosphate (NADPH) oxidase (Figure 1). Deficiency of any one subunit of NADPH oxidase (gp91^phox^, p22^phox^, p47^phox^, p67^phox^, p40^phox^; Table 1), or of other factors that contribute to the respiratory burst pathway (CYBC1/Eros, Rac2-GTPase, G6PD, MPO, GSS; Table 2) may culminate in CGD or a CGD-like phagocyte disorder, reviewed in [54].

CYBB (cytochrome b_558_ beta component) encodes the gp91^phox^ component of NADPH oxidase on the X chromosome, and mutation of this gene accounts for XR disease and 65–70% of all CGD [56]. Most cases of CYBB-related CGD are hemizygous males, although haploinsufficient females with very skewed XCI are also well-recognised, and show predominantly autoimmune phenotypes [45,59,60]. gp91^phox^ is a glycosylated integral membrane protein and the central enzymatic subunit of the NADPH oxidase complex. CYBA (cytochrome b_558_ alpha component) encodes the p22^phox^ subunit, and mutation of this gene is responsible for about 5% of CGD, heritable in an AR manner and equal in severity to XR disease [55,61]. p22^phox^ forms a heterodimer with gp91^phox^, and stable expression of gp91^phox^-p22^phox^ depends on the chaperone protein CYBC1/Eros. More recently described rare mutations of this gene are responsible for a small subset of AR CGD [62,63,64,65,66].

The p47^phox^, p67^phox^ and p40^phox^ subunits form a distinct heterotrimeric complex in the cytosol in resting phagocytes, and together activate NADPH oxidase when the respiratory burst pathway is triggered, e.g., by microorganisms engaged via membrane receptors. NCF1 (neutrophil cytosolic factor 1) encodes p47^phox^, and mutation of this gene accounts for about 20% of all CGD [67,68,69]. NCF1-related CGD is generally mild in severity [55]. NCF2 encodes p67^phox^, and mutation of this gene causes AR disease that is as severe as XR CGD [67,69]. NCF4 encodes p40^phox^, and mutation of this gene causes a rare, mild and atypical form of CGD with more inflammatory clinical manifestations [19,70,71]. Upon encountering phagocytic or inflammatory stimuli, signalling pathways converge on NADPH oxidase to activate the respiratory burst [70,72,73,74]. The complex is also engaged by activated Rac2-GTPase, which enhances its enzymatic activity [72,74]. Mutation of RAC2, therefore, disrupts signalling to the NADPH oxidase complex [75], resulting in defects in ROS production and other neutrophil and lymphocyte functions, which manifest as an autosomal dominant (AD) CGD-like syndrome [4,76,77].

Severe G6PD deficiency can compromise supply of NADPH to the respiratory burst, and manifest clinically as mild CGD [4,73,78]. Because of its relatively high prevalence and XR inheritance, G6PD deficiency is an important differential diagnosis for CGD, especially where other clinical features are suggestive (e.g., haemolytic anaemia) [54,56]. Deficiencies of glutathione synthetase, glutathione peroxidase and glutathione reductase are also associated with inflammatory complications akin to CGD, through the roles of these enzymes in oxidative balance within phagocytes [79]. Of note, deficiencies of other ROS-producing systems, such as the mitochondrial respiratory chain, xanthine oxidases, lipoxygenases, nitric oxide synthases or cyclooxygenases do not typically confer susceptibility to infection [2].

NADPH oxidase transfers electrons to apical molecular oxygen to form superoxide radical, which is then converted to other ROS (hydrogen peroxide [H_2_O_2_] and hydroxyl radicals) at the apical membrane, reviewed in [73]. These ROS serve as substrates for peroxidase enzymes (e.g., myeloperoxidase [MPO]), to produce other reactive molecules, e.g., hypochlorous acid and secondary amines. NADPH oxidase activation furthermore leads to potassium ion influx into the phagosome lumen, which activates microbicidal proteases from the granule matrix [72,74,80]. Within phagocytes, these activities are both directly microbicidal and stimulate inflammatory signalling [81]. MPO deficiency is more common than CGD, with estimated prevalence of 1 in 2000–4000, but is usually asymptomatic or causes a limited immunodeficiency with susceptibility to Candida infections [54,82], though not true CGD.

Overall, neutrophils deficient in the respiratory burst show defective killing of microorganisms by phagocytosis, neutrophil extracellular traps, and other forms of regulated cell death [83,84,85,86]. However, defects of ROS-related inflammatory regulation and other innate immune pathways are thought to be responsible for the hyperinflammatory manifestations of CGD [81,87,88,89,90]. Furthermore, granulomas are believed to arise where microbes fail to be eliminated, or there is persistent dysregulated local cytokine production following microbial sterilisation. The resulting chronic inflammatory infiltrate may organise into a structured granuloma containing lymphocytes and histiocytes [28,91].

## 4. Benefits of Early Diagnosis of CGD

**Figure 2:**
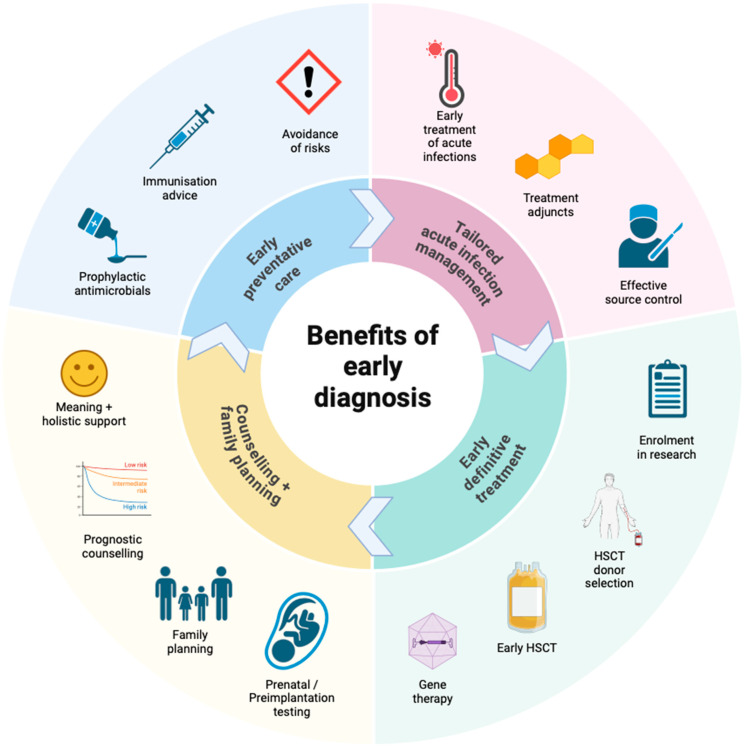
Benefits of early diagnosis in chronic granulomatous disease. HSCT haematopoietic stem cell transplant. Figure made with BioRender^®^.

Like many inborn errors of immunity (IEI), early diagnosis of CGD is important for several reasons, which together support optimal clinical outcomes (Figure 2): (1) early instigation of effective preventative care, (2) appropriate and tailored management of acute infections and complications, (3) early definitive treatment, and (4) early identification of affected family members. Preventative care includes prophylactic antimicrobials (e.g., co-trimoxazole and azole antifungals), active surveillance, immunomodulation (some centres advocate use of interferon-gamma), immunisation advice, and avoidance of risks (e.g., decaying organic matter and high-risk water exposures) [19,24]. Definitive treatment in the form of allogeneic haematopoietic stem cell transplantation (HSCT) is indicated for severe CGD, and is ideally performed early in life, prior to episodes of severe infection, inflammatory complications, alloimmunisation, or organ scarring and dysfunction, which increase the risk of complications associated with HSCT [3,92]. The psychosocial and economic burden of unexplained significant ill-health and its implications for quality of life are also likely to be substantial for patients with undiagnosed severe CGD [22,93,94,95]. Furthermore, survey data highlighting the impact of CGD on psychosocial and functional outcomes for patients and carriers further emphasise the potential benefits of early diagnosis and definitive treatment [96,97,98,99]. For these reasons, prompt recognition and testing are essential.

Achieving a genetic diagnosis of CGD confers several specific additional benefits. Genetic diagnosis can guide prognosis for affected individuals, genetic testing and counselling for family members, and family planning, which may take into account prenatal or preimplantation testing [19]. Genetic diagnosis can also guide appropriate selection of noncarrier HSCT donors among HLA-matched family members, and is essential for directing efforts towards effective gene therapies, which at present remain aspirational and a very active field of research [100,101,102]. Indeed, the potential benefits of genetic diagnosis warrant consideration of DNA banking for affected deceased probands who lack a defined genetic cause for their CGD, as testing methods continue to improve [103]. Prenatal and preimplantation detection of CGD for at-risk pregnancies is now common practice, and methods are reliable for both XR and AR forms of the disorder, particularly where a precise familial mutation is known [19,104,105]. Of note, family and prenatal testing are still possible where a genetic diagnosis is lacking, as conventional functional assays can still be performed on peripheral blood or umbilical vein samples [19,106].

## 5. When to Suspect and Test for CGD

CGD testing is indicated where there is history of repeated invasive bacterial or fungal infections typical for this disorder, tissue granulomas, inflammatory manifestations or complications, especially in the context of a suggestive family history or characteristic histologic or radiologic findings (although the latter are not essential) [107,108] (Box 1). The European Society for Immunodeficiencies (ESID) registry provides working definitions for definite and probable cases of CGD, which reflect the clinical features described above, and require laboratory testing of both the respiratory burst pathway and genetics [109].

Box 1 **Histologic and radiologic findings in CGD.**

            Histologic features of CGD in the lung are nonspecific, but may include: neutrophil-predominant abscesses or micro-abscesses surrounded by histiocytes; granulomas, which may be suppurative, necrotising or non-necrotising; and chronic diffuse inflammation associated with areas of fibrosis [110,111]. Gastrointestinal tract histology findings in CGD involving the gut are also nonspecific, and may be indistinguishable from IBD [112,113,114,115]. These include mucosal granulomas or microgranulomas and focal inflammation in the form of acute cryptitis, crypt abscess and ulceration, as well as chronic lymphoplasmacytic inflammation and disruption of normal mucosal architecture [113,116]. Eosinophils and pigmented macrophages may be present in inflammatory infiltrates, and the latter are reported to be more suggestive of CGD [112,116]. These findings may occur throughout the gastrointestinal tract, but predominantly affect the lower colorectum and perianal area, with sparing of the upper tract and oesophagus [113,116]. Biopsy findings of CGD-related lesions in skin, lymphoreticular system, liver and bladder may share elements of these nonspecific histologic characteristics of acute and chronic inflammation, with granulomas and occasional visualisation of microorganisms [117].
            Radiologic appearances of lung infection in CGD can be varied, and include pulmonary nodules, ground-glass opacities, focal consolidation and masses, with or without cavities, abscesses, effusion or chest wall involvement where infection is invasive [118,119]. Post-infectious and inflammatory complications can also be varied on chest imaging, and may demonstrate fibrosis, septal thickening, bronchiectasis, and emphysema [118,119]. In the gut, patients may again demonstrate nonspecific radiologic findings of wall thickening, dilatation, or mucosal enhancement with a predominance of lower tract and perianal disease (fistulae, fat stranding and abscesses), and endoscopy may or may not show active inflammatory changes [120]. Imaging of CGD lymphadenitis shows nonspecific tomographic appearances of enlarged and contrast-enhanced lymph nodes with or without central necrosis, which sonographically may have thick septations and internal debris; calcification may be seen in chronically inflamed nodes [121]. In the genitourinary tract, wall thickening may be demonstrated in the bladder and ureteric tracts, sometimes with evidence of obstruction (e.g., hydronephrosis) or scarring and calcification [121,122]. Furthermore, complications of CGD in liver, spleen, skin or soft tissue, muscle, bone and central nervous system may demonstrate nonspecific radiologic changes [121].

Typically, an immunological workup in CGD reflects the central deficit in NADPH oxidase [107]. The differential white cell count and immunoglobulin levels are likely to be normal, or nonspecifically altered in the context of intercurrent infection or complication. Complement studies, lymphocyte subsets, vaccine responses and phagocyte morphology are typically normal. Expression of neutrophil cell surface adhesion molecules and tests of chemotaxis are expected to be normal, whereas direct tests of NADPH oxidase function and phagocytic killing are abnormal, as below. Genetic testing is informed by the clinical details and family history, particularly if the familial mutation is already known [108]. Functional and genetic carriership testing should also be offered to adult female relatives of confirmed XR cases, particularly if there are infective or inflammatory manifestations of CGD [45,50,108].

The main differential diagnoses for CGD are disorders of recurrent severe or atypical infection, granulomas and hyperinflammation [107], including cystic fibrosis, severe G6PD deficiency [54], glutathione synthetase deficiency [58], protein kinase C delta deficiency [123,124], leukocyte adhesion deficiency [54], combined or common variable immunodeficiency [91], MyD88/IRAK4 deficiency, hyper-IgE syndrome [125], allergic bronchopulmonary aspergillosis, sarcoidosis, IBD, MPO deficiency [54] and SAPHO syndrome (synovitis, acne, pustulosis, hyperostosis, osteitis). Distinctive phenotypic features may point clinical suspicion in these directions, and prompt alternative methods of diagnostic testing.

## 6. Testing of NADPH Oxidase Function

Defective ROS production mediates the effect of genotype on phenotype in CGD, and diagnosis requires demonstration of a significantly impaired respiratory burst [126]. Most CGD cases exhibit a total lack of NADPH oxidase activity [127]. However, functional assays, which provide quantitative information on residual enzymatic activity, are important for guiding prognosis, because of the well-recognised survival benefit conferred by residual oxidase function [44].

Tests of NADPH oxidase activity can be performed on anticoagulated whole blood or purified leukocytes or neutrophils, reviewed in [108,126,127,128]. Misleading assay results may occur due to pyrogen contamination, sample mishandling and delay in processing, due in part to the short ex vivo lifespan of functional neutrophils and, therefore, strict quality controls are required (e.g., a healthy comparator specimen) [108,126,129,130]. Of note, in certain circumstances, the polymorphonuclear fraction of circulating leukocytes may contain significant numbers of non-neutrophils (e.g., eosinophilia due to atopy or parasitic infection) [126].

Because NADPH oxidase is inactive in resting neutrophils, exogenous stimulation of the pathway is required for functional testing. Serum-opsonised particulate stimuli, such as zymosan (a yeast membrane derivative), *E. coli*, or *S. aureus* provoke the respiratory burst by engagement of Fc-gamma receptor (FC*γ*R) and complement receptor 3 (CR3) on the phagocyte cell surface [108]. Fluid-phase stimuli such as phorbol myristate acetate (PMA) or formyl-methionyl-leucyl-phenylalanine (fMLP) require priming by platelet-activating factor (PAF), and also provoke the respiratory burst [108]. Comprehensive testing of neutrophil function thus requires assays that test multiple different stimuli.

Tests of NADPH function include measurement of oxygen consumption, plate-based assays (ferricytochrome c reduction, lucigenin/isoluminol, Amplex^®^ Red), nitroblue tetrazolium, dihydrorhodamine and dichlorofluorescein assays. Tests of phagocytosis and neutrophil microbicidal function may be carried out in addition as part of a wider workup of a suspected disorder of neutrophil function, but are outside the scope of the current review [127].

### 6.1. Measurement of Oxygen Consumption

Measurement of oxygen consumption using oxygen electrodes can provide a quantitative readout of the respiratory burst pathway [131], but is rarely used in clinical practice because it is time-consuming, technically difficult to set up, and relatively insensitive for diagnosis of CGD [102,108].

### 6.2. Plate-Based Assays

Ferricytochrome c reduction is a colorimetric assay of superoxide production. Ferricytochrome c is added extracellularly and does not permeate cell membranes [126]. Superoxide produced by phagocytes and released to the extracellular space converts ferricytochrome c to the reduced product ferrocytochrome c, which can be measured by spectrophotometry [108,126,132]. The result gives a pooled reading for the whole population of cells tested, and cannot distinguish mixed populations [126]. Having been superseded by other tests, the ferricytochrome c assay is no longer widely used for CGD diagnosis.

Reagents that release energy in the form of light upon reaction with ROS can also be used to measure NADPH oxidase activity by chemiluminescence, e.g., lucigenin or isoluminol [133]. These assays are sensitive and can be carried out relatively quickly on few available cells [126,132,134]. They detect both intra- and extracellular H_2_O_2_, and rely on cellular peroxidases, so may misdiagnose MPO deficiency as CGD [132,134].

Amplex^®^ Red is an alternative chemical probe for H_2_O_2_. The colourless, nonfluorescent dihydroresorufin derivative Amplex^®^ Red is oxidised by H_2_O_2_ to resorufin, which is detectable as red fluorescence, and can be measured using a plate reader [135]. The Amplex^®^ Red assay is also sensitive to small numbers of cells, reliable, and relatively easy to perform, but is not truly quantitative, and again is not routinely used in practice for diagnosis of CGD [124].

### 6.3. Nitroblue Tetrazolium (NBT)

**Figure 3:**
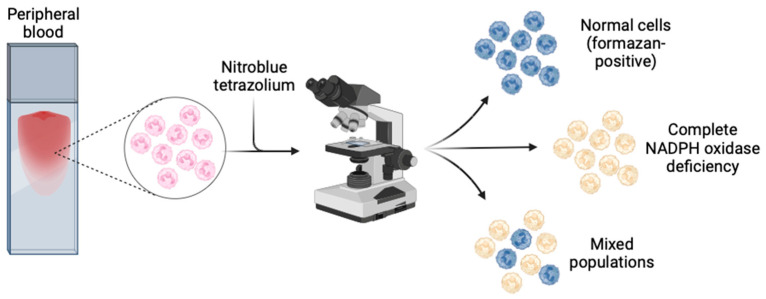
Nitroblue tetrazolium (NBT) test. Figure made with BioRender^®^.

The nitroblue tetrazolium (NBT) assay measures superoxide production, and has been in clinical use for decades for diagnosis of CGD [136]. In this assay, superoxide produced by activated NADPH oxidase converts the pale yellow dye NBT to its dark blue insoluble formazan product, which precipitates within activated phagocytes [126,137] (Figure 3). Quantification can be performed by counting formazan-positive and -negative neutrophils by light microscopy of peripheral blood in a chamber slide, or by spectrophotometric measurement of the solubilised formazan product in neutrophil lysate [108,132]. The assay is theoretically semi-quantitative when carried out by an experienced technician, but is most sensitive for severe CGD, where there is very little to no formazan deposition in any cell [138]. It also has low sensitivity for detecting mixed populations of cells in female carriers haploinsufficient for pathogenic CYBB mutations, and for detecting hypomorphic variants with residual NADPH oxidase function (both milder AR and protein-positive XR CGD) [126]. High-confidence current estimates of sensitivity and specificity of the NBT assay for diagnosing CGD are lacking in published literature. However, because it is quick to perform using nonspecialist materials and equipment, it is particularly useful in low-resource settings, or where a rapid result is required, and local expertise is available [139,140,141].

### 6.4. Dihydrorhodamine (DHR)

**Figure 4:**
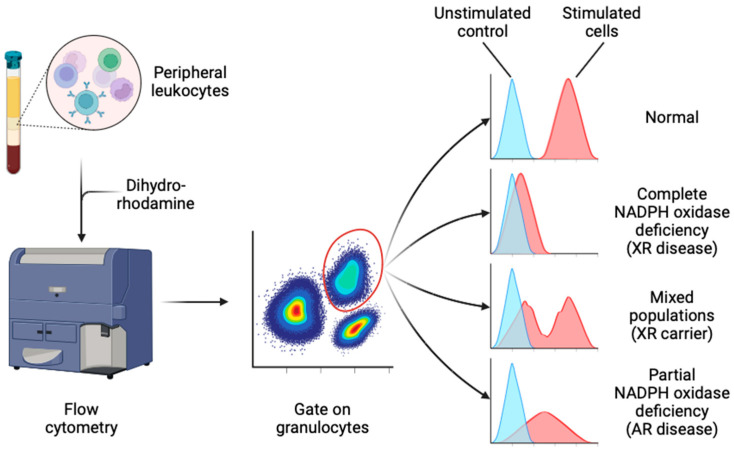
Dihydrorhodamine (DHR) test. Figure made with BioRender^®^.

The dihydrorhodamine-1,2,3 (DHR) assay is considered the current gold standard test of NADPH oxidase activity [126]. In the presence of a peroxidase enzyme (e.g., MPO or eosinophil peroxidase) and stimulated conditions, it allows quantitative measurement of H_2_O_2_ production [142]. DHR freely diffuses into cells and reacts with H_2_O_2_, becoming reduced to rhodamine, which is trapped intracellularly and fluoresces green [142]. The fluorescent signal can be measured in individual cells by flow cytometry [142] (Figure 4). Extracellular addition of catalase minimises background rhodamine signal [126]. Three classic patterns of abnormal DHR result are seen: (1) complete or severe deficiency (rhodamine-positive cells comprise <5% of total neutrophils or <5% of control), (2) hypomorphic or partial deficiency (where the rhodamine signal is uniformly intermediate between unstimulated and stimulated control neutrophils), and (3) mosaic patterns, where a proportion of neutrophils show reduced NADPH oxidase function and the remainder are normal [126,143] (Figure 4).

The DHR assay is highly sensitive, quantitative and relatively easy to perform in facilities with standard flow cytometry capability [126], though this has obvious implications for test availability in low-resource settings [139,140,141]. The DHR assay is especially useful because of its ability to quantify residual NADPH oxidase function, and to discriminate carrier status and quantify donor chimerism post-HSCT [44,50,144,145]. Notably, within XR CGD carriers, the proportion of DHR-positive neutrophils is reported to vary over time [143,146].

False positive DHR results can arise in severe MPO deficiency, which mimics CGD, although superoxide production, and hence ferricytochrome c reduction and the NBT assay, are normal [147,148]. Other false positives can occur in severe G6PD deficiency [78] and SAPHO syndrome [149], where superoxide production is abnormal, and in conditions of acquired neutrophil dysfunction, such as granulocytic ehrlichiosis [150,151], and reportedly after exposure to anti-inflammatory drugs [152,153,154]. The DHR assay cannot detect carriers of AR CGD, because a single functional allele of an autosomal gene is usually sufficient for cellular function. Some residual H_2_O_2_ (but not superoxide) production is reported to persist in NCF1-deficient neutrophils [127,155]. Routine DHR testing is also reportedly not sensitive for the rare cases of CGD caused by mutation in NCF4 [19]. In the latter case, the PMA/fMLP-stimulated DHR response is normal or only mildly reduced, but oxidative responses to particulate stimuli are defective, and killing of certain microorganisms is also impaired [71,127]. In Rac2-GTPase deficiency, PMA-stimulated superoxide production is normal or increased compared to control, whereas fMLP-stimulated chemotaxis and superoxide production are impaired [75,76], along with other non-phagocyte immune defects [2,156]. In CYBC1/Eros deficiency, as expected, there is demonstrable loss of PMA-stimulated respiratory burst measured by the DHR assay due to the lack of expressed gp91^phox^-p22^phox^ [62].

### 6.5. Dichlorofluorescein (DCF)

2′7′-dichlorofluorescein (DCF) and 5,6-carboxy-2′7′-dichlorofluorescein diacetate (CDCF) are alternative fluorescent probes, which can be used in flow cytometric assays of NADPH oxidase function, but are used less commonly in practice than DHR [157].

## 7. Molecular Diagnostics: Protein Assays

Where NADPH oxidase activity is confirmed to be absent by functional assays, protein expression of NADPH oxidase components can be measured to narrow the differential diagnosis of genetic causes of CGD [127]. These assays largely rely on antibodies specific for protein components of NADPH oxidase, and can be carried out alongside other protein assays for suspected disorders of neutrophil function, using anticoagulated blood [108,126]. Protein assays include western blot of neutrophil lysates [108] and flow cytometry-based assays that use surface- or intracellular antibody stains [108,158,159,160,161]. Additionally, antibody-based immunofluorescence and immunohistochemistry for NADPH oxidase subunits have been reported in formalin-fixed, paraffin-embedded tissues, but are not routinely used for CGD diagnosis [162]. It is also possible to detect NADPH oxidase proteins by mass spectrometry, but this is not widely used outside the research space of high-throughput screening for IEI [163,164].

Although western blot is sensitive to detect even small traces of protein, which can be relatively quantified by densitometry, certain caveats complicate the use of this method for diagnostic testing, and require strict quality control and validation [108,126]. Antibody binding and specificity may be sensitive to degradation of blood or tissue samples following collection [162]. Antibodies may be nonspecific to individual NADPH oxidase subunits, but rather recognise the complex as a whole or individual hemi-complexes (gp91^phox^-p22^phox^ or p47^phox^-p67^phox^-p40^phox^) [126,165,166]. As described, gp91^phox^-p22^phox^ expresses as a stable complex integral to the membrane, so isolated deficiency of either subunit alone will not be distinguished from the other solely by protein-based assays [102]. Another caveat is that positive antibody staining does not guarantee that functional protein is present, as intact expression of enzymatically inactive protein will still result in respiratory burst deficiency and CGD [167]. Protein assays may not, therefore, reliably discriminate between genetic diagnoses, although the result can be suggestive when considered in the context of the inheritance pattern and clinical features [165]. Of note, flow cytometry-based assays using antibody probes may distinguish mixed populations in XR carrier or mosaic states, and be useful in these cases [127].

Cell-free NADPH oxidase assays have also been developed to distinguish biochemical deficits in cytosolic and membrane-bound NADPH oxidase components [168]. Purified proband neutrophil membranes are mixed with healthy neutrophil cytosol (or vice-versa), and incubated with NADPH oxidase substrates and activators. Oxidase activity is then measured by superoxide production or oxygen consumption [168]. Furthermore, the nonfunctioning CGD gene may be identified by restoration of the biochemical deficit in EBV-transformed B cells from the proband, by transducing a retroviral vector to express wild-type cDNA for each of the NADPH oxidase components in turn [169]. Again, however, these assays are not routinely used in practice for diagnosis of CGD.

## 8. Molecular Diagnostics: Genetic Testing

Confirmation of a CGD diagnosis following positive functional testing is established by identifying a pathogenic variant in one of the CGD-related genes that encode or permit assembly or function of NADPH oxidase [19]. Algorithms that incorporate different genetic and genomic techniques into a standardised workflow for the investigation of CGD have been proposed [170], and reporting of genetic results generally involves classifying identified variants according to the likelihood that they are responsible for the clinical phenotype (Box 2). Genetic testing methods, reviewed in [19], generally include: (1) gene-targeted testing in single- or multi-gene panels performed by Sanger or next-generation sequencing (NGS) techniques; (2) comprehensive genomic testing, including whole exome (WES) and whole genome sequencing (WGS); (3) copy number variant (CNV) analysis, e.g., by multiplex ligation-dependent probe amplification (MLPA) or comparative genomic hybridisation (CGH) arrays; and (4) ancillary methods, e.g., single nucleotide variant (SNV) arrays, mRNA size and sequence analysis, deep amplicon sequencing, and optical gene mapping [171] (Box 3). The choice of genetic techniques applied depends on the phenotype and clinical suspicion (clinical details, family history, and functional assay results), and confirmatory testing should be applied as standard within genetic testing pipelines. Family history is essential to guide analysis and interpretation of sequencing data (e.g., by application of bioinformatic filters based on inferred modes of inheritance), and testing is far easier where the familial mutation is already known [172]. For genetic testing, EDTA blood is preferred, and genomic DNA or total RNA can be extracted from peripheral blood mononuclear cells (PBMCs) [108].

Box 2 **Interpretation of genetic variants.**

          Genetic variants, regardless of the method used for their detection, are categorised broadly by the likelihood that they are responsible for an individual’s clinical phenotype. These are summarily termed categories 1 to 5 on the joint recommendation of the American College of Medical Genetics and Genomics and the Association for Molecular Pathology [173]. In brief, variants that have previously been confirmed as benign in published literature, curated databases of genetic variants, or unpublished in-house laboratory data are termed category 1 “benign” variants. Those that are predicted by computational (in silico) or other means to be benign are termed category 2 “likely benign” variants. “Variants of uncertain significance” (VUS), which differ from the reference genome but cannot be confirmed or ruled out as responsible for the phenotype in question are classified as category 3 variants. Category 4 “likely pathogenic” variants are predicted computationally or otherwise to be pathogenic, but have not been conclusively proven as such in existing data sources, as listed above. Lastly, category 5 “pathogenic” variants are confirmed to be disease-causing in published literature or databases.          For the purposes of clinical practice, category 4 and 5 variants are generally considered diagnostic positive, and category 1 and 2 variants are considered negative. By consensus, category 3 VUS should not be used to guide clinical decision-making for affected individuals [173]. Interpretation of VUS can be aided by trio and segregation studies in individual cases, and functional testing of suspected variants expressed in in vitro systems within experienced research settings [174]. Different labs and genomic testing pipelines may manage VUS differently, e.g., in reporting pre-test probability of finding VUS in their panel, or may not report VUS at all [172]. Of note, reference databases for variant classification are gradually refined over time, and most VUS are eventually reclassified into other categories, prompting many genomic laboratories to periodically re-analyse historic clinical sequencing data, and issue supplementary reports where reclassification impacts the interpretation of genetic findings in individual cases [175]. In the context of CGD, clinical symptoms alone are insufficient to confirm pathogenicity of a novel CGD gene variant without first demonstrating an oxidative burst defect [108].

Box 3 **Genetic and genomic testing methods: uses and limitations.**

1.Gene-targeted testing
        Gene-targeted testing requires clinicians to determine *a priori* which are the likely genes involved in an individual’s presentation, and include “off the shelf” gene panels (including those commonly associated with broad phenotypes or recognisable clinical syndromes with heterogeneous genetic aetiologies) and custom-designed panels (the clinician selects genes for analysis) [172]. Multigene panels vary by laboratory in terms of gene coverage and technique, and assays may include sequence and CNV analysis, as well as ancillary tests to cover regions that are problematic for conventional NGS (e.g., highly homologous pseudogenes, deep intronic pathogenic variants, and expanded nucleotide repeats) [172].        Gene panels have certain advantages over comprehensive testing. Included genes have usually been specifically targeted and validated by the laboratory, allowing greater confidence in complete sequencing of the genes of interest, and often include relevant targeted noncoding regions, which may be missed by exome sequencing [172]. Choosing a narrow phenotype-focussed panel also has the advantages of streamlined analysis, reduced costs, quicker turnaround time, and limited identification of VUS and incidental variants in genes unrelated to the disease phenotype, which may cause further clinical uncertainty or be clinically unactionable [172].
2.Comprehensive genomic testing
        Comprehensive genomic testing does not require the clinician to determine which are the likely genes involved in the individual’s presentation. Comprehensive testing is most likely to be useful where the clinical features are not suggestive of a known genetic condition decipherable from the phenotype alone, or are suggestive of several genetic conditions at once, which are not included within one multigene panel [172]. Comprehensive testing may also be appropriate where the phenotype is poorly defined (e.g., at earlier stages in the diagnostic evaluation of an acutely and severely unwell child), or where a rare disorder is suspected, which would not routinely be tested for within available multigene panels [172]. Mitochondrial DNA sequencing can also be requested as a separate test where mitochondrial disorder is suspected.
        Whole exome sequencing (WES) and whole genome sequencing (WGS) can reliably detect missense or nonsense variants, and small indels (<50 bp) within nonrepetitive coding DNA that are rare in the population and previously reported as pathogenic [172]. WES examines the approximate 180,000 protein-coding segments of the genome (exons), which comprise 1–2% of the genome and account for the majority of recognised pathogenic variants [172]. About 95% of the exome can be sequenced with current NGS methodologies [176,177]. WGS examines the entirety of the approximate 20,000 genes, noncoding RNAs, intronic and intergenic regions of DNA [172]. WGS has the advantages of (1) being able to detect intronic or intergenic variants not covered by WES, (2) simpler sample preparation methods (e.g., no need for sequence enrichment for coding regions), and (3) being able to identify structural variants and chromosomal breakpoints in noncoding regions [172].
        Certain regions still remain elusive to standard NGS technologies, and the specific methodology used to target these will vary by laboratory. WGS is slower and more expensive than WES, and yet the majority of pathogenic variants identified by WGS are located within exons [172]. Rapid evolution of WES/WGS sequencing and analysis methods in recent years precludes precise estimation of their diagnostic accuracy for inborn errors of immunity (IEI), but reported estimates of sensitivity range from 83 to 100%, and of specificity range from 45 to 88% [178]. Of note, where more genes are tested at once, there is greater likelihood of finding VUS and incidental pathogenic variants, which may pose difficulties for clinical interpretation and management [179].
3.Copy number variant analysis and ancillary methods
        Chromosomal microarrays (CMAs) detect copy number variants (CNVs), which may range in size from 1 kilobase to multiple megabases or even whole chromosomes, and therefore contain 0, 1 or many genes or parts thereof [179]. Microarrays include oligonucleotide comparative genomic hybridisation (CGH) arrays, single nucleotide variant (SNV) arrays, or combinations of these technologies, and can be designed to test at genome-wide scale or in targeted regions with appropriate resolution [172]. CMAs are more sensitive than traditional karyotyping for CNV detection, and can even be designed at exon-level resolution for specific genes [180,181].        Ancillary methods may be used to supplement genetic or genomic results obtained by conventional means. For example, the impact of predicted pathogenic intronic variants (especially those outside of the core or essential splice site) may be confirmed by RNA analysis, as missplicing may lead to complete or partial exon skipping or inclusion of intronic sequence in mature mRNA, with deleterious consequences [170].

## 9. Mutations in CGD

The heterogeneity of clinical phenotypes observed in CGD is reflected in the variety of mutations identified in responsible genes. Multigene panels for suspected IEI routinely include the core genes responsible for CGD (CYBB, CYBA, NCF1, NCF2, NCF4 and CYBC1), and should also include other genes of interest responsible for important differential diagnoses (e.g., CFTR, G6PD, RAC2 and GSS). These genes should all be captured by WES/WGS methods, but it is important to confirm that they are included within the analysis when CGD is suspected.

CYBB mutations range from single nucleotide variants (missense, nonsense) and small insertions and deletions (indels), to deletions of several megabases, and may involve exons or splice sites [56]. Large insertions are relatively less common, and are usually caused by duplications or transposable elements [56,127,182]. Some CYBB mutations result in complete absence of gp91^phox^ protein expression, and complete deficiency of NADPH oxidase function, termed the “Xb^0^ phenotype” [127]. Some CYBB mutations result in a degree of residual functional protein expression (e.g., amino acid substitutions or small in-frame indels, and splice site mutations that leave some residual normal mRNA), termed the “Xb^−^ phenotype” [127]. These comprise mostly pathogenic missense variants in the region encoding the N-terminal 309 amino acids, and tend to confer relatively better prognosis [44]. Variants affecting the C-terminal portion affect cofactor and substrate binding domains, and render the expressed protein largely nonfunctional with relatively poorer prognosis [44]. Yet, other CYBB mutations result in expression of normal levels of enzymatically inactive protein, termed the “Xb^+^ phenotype” [127]. Of note, a distinct spectrum of hypomorphic missense mutations in CYBB, which specifically impair NADPH oxidase function in macrophages and B cells (rather than granulocytes), leads to XR Mendelian susceptibility to mycobacterial disease (MSMD) [183,184].

About 15% of CYBB mutations arise de novo, where maternal leukocyte DNA does not contain the mutant allele, but this does not exclude the possibility of maternal somatic mosaicism, where some or all of the maternal gonadal or germ cells harbour the mutation. As such, if maternal testing is negative for the mutation, there may still be a risk that further offspring will be affected [13]. As described earlier, skewed XCI is also responsible for clinical features of overt CGD in a subset of female carriers of pathogenic CYBB variants [11,59]. Where this is suspected, the standard HUMARA assay for XCI may be used, which assesses epigenetic silencing of X-chromosome genes [185,186]. Novel methods, which use NGS to directly assess differential transcription of X-chromosome genes have also been developed more recently to assess for skewed XCI [170]. Individuals with suspected XR CGD and clinical manifestations of McLeod syndrome (neuromuscular abnormalities and haemolytic anaemia) should undergo CMA to detect larger deletions within the X chromosome [187,188]. CMA with adequate resolution to identify the deletion boundary can define whether there is loss of implicated genes contiguous to CYBB in these cases, e.g., XK (Kx blood group antigen), RPGR (retinitis pigmentosa), DMD (Duchenne muscular dystrophy) and OTC (ornithine transcarbamoylase) [187,188].

NCF1 has two neighbouring paralogous pseudogenes >99% identical in sequence to NCF1, which pose a particular challenge to genetic diagnosis by conventional sequencing [189]. Both pseudogenes harbour a two-nucleotide GT deletion at the beginning of exon 2, which leads to frameshift and a premature stop codon, resulting in absent expression of active p47^phox^ protein [127,190]. Loss of the functional NCF1 gene may occur due to unequal crossover between the intact gene and either pseudogene during meiosis [189,191]. NCF1-ΔGT accounts for about 90% of all p47^phox^-deficient CGD, and compound heterozygotes with other NCF1 mutations have also been reported [55]. To detect these variants, PCR and sequencing may rely on primers that discriminate between NCF1 and its pseudogenes [108], or on methods that can demonstrate a 2 bp length discrepancy between amplified fragments of the NCF1 gene or pseudogene [192,193]. mRNA and protein assays may also have a role in confirming pathogenicity of detected NCF1 variants [194]. Of note, another distinct group of NCF1 variants is reported to be associated with autoimmune and autoinflammatory disorders without the other features of CGD [195].

Mutations in CYBA and NCF2 include indels, missense, nonsense and splice site variants [55]. Most lead to complete absence of protein expression, but a few reported mutations lead to decreased expression of functional protein, and thus reduced NADPH oxidase activity [196,197]. There are few reported cases of NCF4 deficiency, but described variants include missense and nonsense mutations affecting exons, introns or essential splice sites, as well as in-frame deletions [70,71,167]. Mutations of CYBC1 result in loss of expression of the gp91^phox^-p22^phox^ protein complex, but levels of CYBB and CYBA mRNA are preserved [63,64]. Reported mutations of RAC2 include missense variants, which encode an inactive protein unable to bind GTP [4,75,76,156]. Only severe forms of G6PD deficiency result in such scarcity of NADPH as to phenocopy the infection susceptibility of milder forms of CGD [54]. As such, these mutations (mostly missense) comprise only a small subset of all recognised G6PD mutations [56]. Although a theoretical possibility, very skewed XCI in haploinsufficient carriers of G6PD deficiency may also result in CGD features in females.

## 10. Limitations of Genetic Testing for CGD

Broadly, challenges in genetic diagnosis of CGD reflect both technical and biological factors common to other genetic disorders. Where sequence variants are not detected by genetic or genomic methods, there are several potential explanations.

Specimen type and handling can impact the yield and quality of nucleic acid, and thus sequencing performance, e.g., NGS read depth and accuracy of base calling, although standard quality controls should identify these issues early [108]. NGS technologies are subject to technical variables that affect diagnostic sensitivity, including the sequence enrichment method used for exome sequencing, use of paired- versus single-end read methods, and use of short- versus long-read technologies [172]. It is worth noting that standard NGS techniques cannot detect variants that influence gene expression without changing DNA sequence (e.g., epigenetic or imprinting errors, and uniparental disomy), and do not reliably detect variants in highly repetitive regions of DNA that are difficult to sequence (e.g., nucleotide repeat expansions or contractions), variants involving a gene that is highly homologous to other gene family members or pseudogenes (e.g., NCF1), and variants that are somatically acquired or mosaic (i.e., only present in a small percentage of cells, such that base calls do not pass quality thresholds).

Gene panels or WES may not detect pathogenic variants in regions (e.g., introns or regulatory regions) not covered by the sequencing method used. Conventional sequence analysis alone is also poorly sensitive for structural variants and large whole-exon or whole-gene deletions or duplications (e.g., McLeod syndrome), and these types of mutation require alternative methods of CNV analysis or high-resolution karyotyping [19]. However, creative new methods based on NGS and Sanger sequencing have the potential to address some of these historic challenges in diagnosing CGD and investigating skewed XCI and mosaicism [170].

It is worth emphasising that genetic and genomic testing workflows depend upon the quality of the clinical information and family history provided by the requesting clinician, and diagnostic sensitivity may be affected if these are inadequate. Interpretation of the significance of detected variants is also subject to the limitations of existing literature, databases and bioinformatic prediction tools [173] (Box 2). Notwithstanding technical factors, the proband may also truly not have a pathogenic variant in the tested genes, and an alternative explanation for their clinical syndrome should be sought. Where clinical suspicion for CGD remains high, this warrants close inspection of the methods employed, and periodic re-analysis of existing data to take account of updated bioinformatic methods and reference databases. In some cases, following discussion with genomic service providers, repeat testing may be warranted in due course to account for technical improvements, which might improve diagnostic yield.

Because CGD is currently understood to be a rare diagnosis, clinical awareness and suspicion may not be sufficient to prompt testing for CGD in the first place, and awareness of the need to test presents a significant challenge to the timely diagnosis of this disorder. The availability of genetic and genomic testing is also restricted in resource-limited settings, which remains one of the ongoing challenges to identifying and helping those CGD cases whose need is greatest [198,199,200]. Even in high-resource settings such as the UK, significant reporting backlogs in centralised genomic testing services have the potential to contribute to significant delays in diagnosis and treatment [201].

## 11. Screening

Routine newborn population screening for CGD is not commonplace, despite the potential benefits of early diagnosis [163]. This is due at least in part to the infeasibility of a CGD-relevant screening assay, which can be run in parallel with other tests, although potential assays have been proposed using mass spectrometry for protein detection in newborn dried blood spot samples [164]. Screening of asymptomatic individuals is thus usually restricted to family members of known CGD probands. However, there is growing interest in the use of genomic tests for newborn screening [202,203], and it is timely that five CGD-related genes are included in those to be tested in the Generation Study of newborn genomic screening currently being piloted in sites across the UK [204]. This furthermore emphasises the need for reliable and accurate confirmatory diagnostic testing for screen-positive individuals identified prior to onset of any infective or other complications.

## 12. Outlook

Within the realm of testing for CGD and other genetic disorders, gene panels, sequencing technologies, bioinformatic tools, reference databases and diagnostic sensitivity are likely to vary between laboratories and over time. As services evolve to keep pace with novel developments, we are heartened that functional and genetic testing for CGD continues to improve. We hope that these improvements will help to overcome the diagnostic challenges in CGD discussed here, and facilitate wider access and uptake of testing. Particularly with the expansion of screening into younger age groups using genomic testing in infancy, the future for early diagnosis and definitive treatment of CGD looks much brighter than it did before.

## Figures and Tables

**Table 1:** Genetic causes of chronic granulomatous disease (CGD): NADPH oxidase components.

**Gene symbol**	CYBB	CYBA	NCF1	NCF2	NCF4
**NCBI gene ID**	1536	1535	653361	4688	4689
**Gene name**	Cytochrome b_558_ beta	Cytochrome b_558_ alpha	Neutrophil cytosolic factor 1	Neutrophil cytosolic factor 2	Neutrophil cytosolic factor 4
**Gene location**	Xp21.1-p11.4	16q24.2	7q11.23	1q25.3	22q12.3
**Exon count**	14	6	11	20	12
**Protein**	gp91^phox^/NOX2	p22^phox^	p47^phox^	p67^phox^	p40^phox^
**Protein location**	Specific granule membrane, plasma membrane	Specific granule membrane, plasma membrane	Cytosol, cytoskeleton	Cytosol, cytoskeleton	Cytosol, cytoskeleton
**Frequency**	65–70%	5%	20%	5%	Rare
**Inheritance**	XR	AR	AR	AR	AR
**Clinical severity**	Severe Early onset	Severe Early onset	Mild Late onset	Severe Early onset	Mild Atypical
**Mutations**	Missense Nonsense Missplicing Large or small deletions Insertions Duplications Transposable elements	Insertions Deletions Missense Nonsense Missplicing	Unequal meiotic crossover events with neighbouring pseudogenes Missense Nonsense Insertions Deletions Missplicing	Insertions Deletions Missense Nonsense Missplicing	Insertions Deletions Missense Nonsense Missplicing
**Pickup on sequence analysis**	85%	85%	75%	85%	90%
**Pickup on deletion/duplication analysis**	15%	15%	25%	15%	10%

Abbreviations: X-linked recessive, XR; autosomal recessive, AR. Reviewed in [55,56].

**Table 2:** Rare genetic causes of chronic granulomatous disease (CGD) or related disorders: non-NADPH oxidase causes.

**Gene symbol**	CYBC1	RAC2	G6PD	MPO	GSS
**NCBI gene ID**	79415	5880	2539	4353	2937
**Gene name**	Cytochrome b_558_ chaperone 1	Rac family small GTPase 2	Glucose-6-phosphate dehydrogenase	Myeloperoxidase	Glutathione synthetase
**Gene location**	17q25.3	22q13.1	Xq28	17q22	20q11.22
**Exon count**	8	8	14	12	15
**Protein**	CYBC1/Eros	Rac2-GTPase	G6PD	MPO	GSS
**Protein location**	Endoplasmic reticulum	Cytosol	Cytosol	Granule lumen	Cytosol, nucleus, mitochondria
**Frequency**	Rare	Rare	Severe deficiency is rare	Common but unlikely to cause CGD-like features	Rare
**Inheritance**	AR	AD	XR	AR	AR
**Clinical severity**	Few cases described	Few cases described	Spectrum from mild to severe	Asymptomatic or susceptible to *Candida*	Spectrum from mild to severe
**Mutations**	Nonsense Missense Missplicing	Nonsense Missense	Deletion Missense	Nonsense Missense Missplicing Deletions	Indels Nonsense Missense Missplicing
**Notes**	Also regulates expression of proteins other than gp91^phox^-p22^phox^, e.g., P2X7	Also associated with abnormalities of neutrophil chemotaxis and lymphocyte function	Mild cases: relatively common but lack CGD-like features Severe cases: generally mild CGD features	Rarely solely responsible for immunodeficiency	Mild cases: haemolytic anaemia only Moderate: metabolic acidosis Severe: progressive neurological dysfunction and infection susceptibility

Abbreviations: X-linked recessive, XR; autosomal recessive, AR; autosomal dominant, AD. Reviewed in [55,56,57,58].
